# Laboratory Study of the Displacement Coalbed CH_**4**_ Process and Efficiency of CO_**2**_ and N_**2**_ Injection

**DOI:** 10.1155/2014/242947

**Published:** 2014-03-06

**Authors:** Liguo Wang, Yuanping Cheng, Yongkang Wang

**Affiliations:** ^1^National Engineering Research Center for Coal & Gas Control, China University of Mining & Technology, Xuzhou 221116, China; ^2^College of Safety Science and Engineering, Henan Polytechnic University, Jiaozuo 454000, China

## Abstract

ECBM displacement experiments are a direct way to observe the gas displacement process and efficiency by inspecting the produced gas composition and flow rate. We conducted two sets of ECBM experiments by injecting N_2_ and CO_2_ through four large parallel specimens (300 × 50 × 50 mm coal briquette). N_2_ or CO_2_ is injected at pressures of 1.5, 1.8, and 2.2 MPa and various crustal stresses. The changes in pressure along the briquette and the concentration of the gas mixture flowing out of the briquette were analyzed. Gas injection significantly enhances CBM recovery. Experimental recoveries of the original extant gas are in excess of 90% for all cases. The results show that the N_2_ breakthrough occurs earlier than the CO_2_ breakthrough. The breakthrough time of N_2_ is approximately 0.5 displaced volumes. Carbon dioxide, however, breaks through at approximately 2 displaced volumes. Coal can adsorb CO_2_, which results in a slower breakthrough time. In addition, ground stress significantly influences the displacement effect of the gas injection.

## 1. Introduction

The warming of the climate system can very likely be attributed to the increase of greenhouse gases in the atmosphere, from 278 ppm before the industrial revolution to 396 ppm in 2013 [1]. Many nations have begun active measures to decrease CO_2_ emissions. There are several methods that can be used to achieve this goal, namely, reducing energy consumption at the production level through more efficient technologies and at the consumption level through changes in lifestyle by extending the use of zero-CO_2_ emission technologies such as renewable energies and nuclear energy and by capturing the CO_2_ produced and storing it deep underground, separated from the atmosphere. Geological disposal is regarded as a feasible and effective approach to sequester CO_2_, and depleted hydrocarbon reservoirs, deep unminable coal, saline aquifers, and the deep ocean appear to be suitable sites for permanent CO_2_ storage [2–4]. Because of the enhanced gas recovery possibilities, coalbeds are one of the most attractive options of all the underground CO_2_ storage possibilities because of the dual benefits of CO_2_ storage and the recovery of coalbed methane (CBM). The revenue of methane production can offset the costs of capture, compression, transportation, and storage of CO_2_ [5].

Conventional primary recovery of methane, which is performed by pumping out water and depressurizing the reservoir, allows the recovery of 20–60% of the methane originally present in the reservoir [6, 7]. This process is called enhanced coalbed methane recovery (ECBM), which is a technique that is under investigation as a possible approach for the geological storage of CO_2_ in the capture and storage system. ECBM recovery is not yet a mature technology in spite of the growing number of pilot and field tests worldwide that have shown its potential and highlighted the attendant difficulties [8–11].

Currently, during ECBM, high-pressure gas goes through hundreds of meters or even kilometers into the coal seam from the wells. It is then discharged from the other wells, which form a complex network. The flooding by injected gas and displacement flows in the coal seam become quite complex. Real-time monitoring of the flow rate and pressure parameters is difficult. Therefore, scholars use physical simulations to study ECBM technology.

ECBM core flooding experiments are a direct way to observe the gas displacement process and efficiency by inspecting the produced gas composition and flow rate. These experiments involve placing a coal sample in a pressure cell and establishing an initial methane content by holding a methane pressure until the adsorption has equilibrated. Gas, for example, CO_2_, is then injected at one end of the sample, and outflow is allowed to occur at the opposite end via a backpressure regulator. Monitoring gas rates and composition provides information on the enhanced gas drainage process.

Experimental research in the aforementioned field has been carried out since the early 1980s. Fulton et al. and Reznik et al. conducted CO_2_ floods of both dry- and water-saturated coal cores that were initially saturated with methane. The results indicated that CO_2_ injection can effectively displace the methane [12, 13]. Parakh presented a systematic approach to performing one-dimensional slim tube displacement for enhanced coalbed methane recovery [14]. Displacement experiments with pure N_2_, CO_2_, and various mixtures were presented. The experiments analyzed the influence of injection pressure and injection rate on the methane recovery and evaluated the influence of water on the CH_4_-CO_2_ exchange process [15]. Jessen et al. conducted displacement experiments with pure CO_2_, N_2_, and various mixtures using a coal briquette in which coal particles were formed into a coalpack by pressing ground coal into cylindrical shapes [16]. Connell et al. reported a study of core floods at two pore pressures, 2 MPa and 10 MPa, and used either nitrogen or flue gas (90% nitrogen and 10% CO_2_) flooding of core samples initially saturated with methane [17]. Dutka et al. presented a study of CO_2_/CH_4_ exchange sorption in a coal briquette. A briquette with a porosity of 8.3%, a diameter of 0.096 m, and a length of 0.280 m was used. It was observed that a pore pressure depression moving along the briquette accompanies the exchange sorption [18, 19]. Zhou et al. conducted a laboratory and numerical simulation of ECBM with pure N_2_ or CO_2_ as injectants. The results showed that the N_2_ breakthrough occurs earlier than CO_2_ breakthrough [20].

At present, physical simulations mainly focus on competitive adsorption tests with fine-grained coal particles or coalpacks with dimensions measured in millimeters or less, or displacement experiments using loose coal (permeability greater than 10 × 10^−15^ m^2^) and small coal cores, none of which accurately reflects the displacement mechanism and process [12–16].

Therefore, we conducted injection and recovery experiments in the laboratory on large specimens to simulate scenarios of CO_2_ injection and CH_4_ recovery in a coalbed. Coal briquettes of 300 × 50 × 50 mm were carefully prepared. The change of the pore pressure in the process of displacement, gas composition, and concentration was dynamically monitored. The experiments were conducted to research the influence of injection pressure and crustal stress on methane recovery.

## 2. Experimental Methodology

### 2.1. Experimental Principle

**Figure 1:**
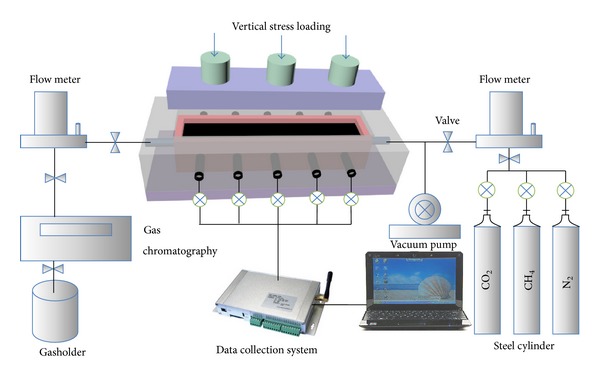
Schematic diagram of experimental setup.

The results of the ECBM process are a combination of the effects of adsorption-desorption, diffusion, convection, and convective dispersion. Adsorption/desorption of a gas can cause swelling/shrinkage of the coal matrix, influencing the permeability, which makes the displacement process more complex as it is coupled with the stress. This means that the real displacement process cannot be simulated in the laboratory. Physical experiments using large coal samples are expensive and time consuming. To demonstrate the dynamic flooding process, a two-dimensional flooding experimental system under stress conditions was constructed here. The experimental schematic diagram is shown in Figure 1.

### 2.2. Experimental Apparatus

Figure 3 shows a schematic representation of the experimental apparatus used in ECBM tests. The apparatus has seven parts as follows: briquette holder, mechanical loading system, injection system, vacuum-pumping system, gas-sampling system, gas-measuring system, and gas-composition analysis system.

(*1) Briquette Holder*. The enclosure walls of the briquette holder are Q235 40 mm thick steel plate. The floor is made of 30 mm thick Q235 steel plate. The cover plate is an activity, which is used for vertical stress loading during the experiment. The experimental cavity size of the cabinet is 300 × 70 × 70 mm. The briquette holder can withstand gas pressure of 6 MPa, which meets the requirements of the experimental pressure.

*(2) Mechanical Loading System*. During the experiment, the vertical load stress is provided by the press machine. It is considered formation pressure. The range of the press is 0–238 MPa.

*(3) Injection System*. The gas injection system consists of cylinders, a compression system, control valves, and pipeline. The control valves are the main valve and pressure-relief valve (pressure ranges from 0 MPa to 16 MPa). The main valve displays the tank pressure. The pressure-relief valve is used to control injection pressure. The maximum output pressure can be up to 10 MPa.

*(4) Vacuum-Pumping System*. The vacuum-pumping system mainly consists of a JZJX30-4 Roots vacuum pump. Coal can be evacuated to a vacuum of <10^−5^ MPa. After checking the tightness of the connections of the displacement apparatus with the Roots vacuum pump, the degassing gas system is connected by turning off the vacuum pump's air communication valve. The degassing time was not less than 48 h. At the end of the degassing process, the vacuum pump was stopped, so it ceased to communicate with the atmosphere.

*(5) Constant Temperature System*. The briquette holder was placed in a water bath that controlled the experimental temperature, maintaining a constant temperature throughout the experiment. The temperature of this experiment was 303 K.

*(6) Gas-Measuring System*. The gas-measuring system measures the injection and effluent gases. The range is 20~200 mL/min. An optical LXI-B7-type flow meter and magnetic levitation LXI-B-type measured the effluent gas. The range of the LXI-B7 is 100~1000 mL/min, and the range of the LXI-B is 10~100 mL/min. Each flow meter was equipped with data acquisition software, so a computer can collect the instantaneous flow rate and total flow at different time intervals.

**Figure 2:**
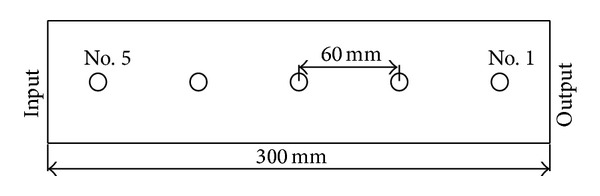
Map of measuring pressure and sampling point.

**Figure 3:**
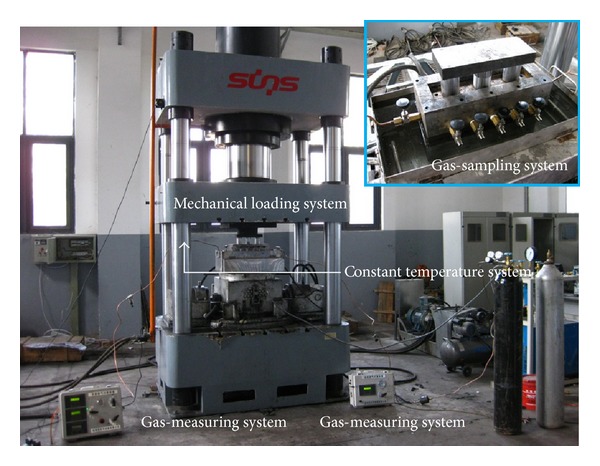
Schematic of experimental apparatus for displacement experiments.

*(7) Gas-Sampling System*. The briquette holder contained six gas sampling points. The distances of the sampling points no. 1–no. 5 from the output of the briquette (no. 0) were as follows: 30 mm, 90 mm, 150 mm, 210 mm, and 270 mm, which are shown in Figure 2. Every sampling point included a 3-way valve that connected to a foil bag used to collect the gas. The arrangement of the manometers is the same as that of the sampling points.

*(8) Gas-Composition Analysis System*. Gas composition was measured with a GC-4000A gas chromatograph. The effluent gas from the flow meter was then sent to a gas analyzer to determine the fraction of each gas species in the effluent mixture.

### 2.3. Experimental Procedures

(1) Place the coal sample into the briquette holder and set the bath temperature to 25°C.

(2) Vacuuming of the sample: the displacement device is connected to the vacuum pump. Due to the large experimental cavity space, the vacuuming time is not less than 48 h.

(3) Injection of methane: after purging the tube, methane is injected at the desired injection pressure for the experiment, with one end of the experimental setup closed. The flow meter is used to determine the amount of methane injected. Injection should be continued for at least 24–48 hours even if the system has stabilized. This is done to make sure that methane not only remains in a free state but also is adsorbed on the surface of the coal.

(4) Injection of carbon dioxide: once the setup is completed, the gas cylinder is turned on, and the injection pressure is adjusted automatically depending on the reducing valve. In general, the injection pressure is more than the original balance pressure of coal specimens.

(5) Measurements: the following parameters are recorded during the injection period for the analysis and interpretation of results:

- the mass of CO_2_ injected into the briquette,
- the mass of the CO_2_-CH_4_ mixture flowing out of the briquette,
- the concentration of the CO_2_-CH_4_ mixture flowing out of the briquette,
- pore pressure changes along the briquette.

(6) Ending the experiment: the experiment is terminated when steady-state concentration conditions are achieved, that is, when the outflow concentration and rate are equal to the inflow. The next experiment was carried out repeating steps (1)~(6).

### 2.4. Sample Description

The sample used for these experiments was from the Yaojie coalfield in China. The details of the samples are shown in Table 1.

Prior to forming the briquette, the coal material was ground to a granularity of 0.2~0.25 mm. To prevent the gas from directly penetrating through the pore between the experimental enclosure wall and the coal wall to the outlet during the gas displacement process, the inwall of the briquette holder, baseplate, and underside of the bearing plate are coated with 10 mm sealant. After 15 days, the sealant was completely solidified. We put the pulverized coal and a small amount of distilled water into the cavity in the body and artificially compact the pulverized coal. The briquette holder was then placed on the press work surface.

Brown and Hoek summarized the research on the in situ stress measurements by the change rule of vertical stress *σ*_*v*_ with depth *H* in various countries as follows [21]:

(1) $$\begin{array}{c} \sigma_{v}=0.027H. \end{array}$$

**Figure 4:**
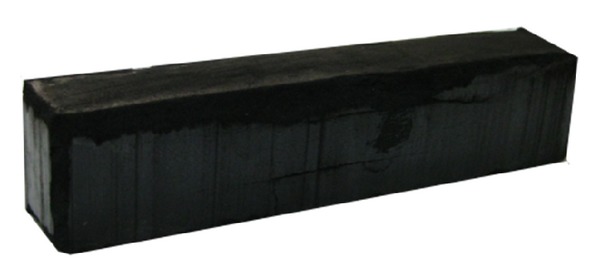
Physical map of the coal briquette.

Based on the fitting formula, we select 14 MPa as vertical stress. The pulverized coal was pressed with a load rate of 1 kN/s to 300 kN, and the force is held at 300 ± 5 kN for 40 minutes. A representative image of the coal briquette is shown in Figure 4.

## 3. Experimental Results

### 3.1. Experimental Schemes

This paper focuses on the displacement coalbed CH_4_ process and efficiency of CO_2_ and N_2_ injection under different gas injection pressures and different stress conditions. The experimental conditions are described in Tables 2 and 3.

### 3.2. Experimental Results****

#### 3.2.1. N_2_-ECBM Experiments

**Figure 5:**
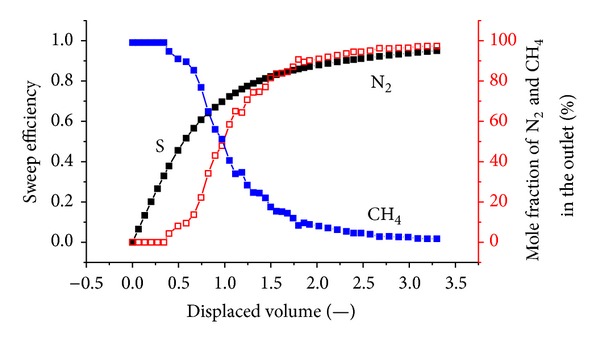
The sweep efficiency and concentrations of produced gas against displaced volume at 1.5 MPa.

Figure 5 shows the sweep efficiency and concentrations of produced gas against displaced volume with a pressure of 1.5 MPa. Prior to N_2_ injection, 9.7 L of CH_4_ was injected prior to equilibrium. Then, N_2_ injection was carried out. The total volume of injected N_2_ is 31.8 L; the outlet volume of the exhaust gas is 36.04 L, including 9.1 L of CH_4_ and 26.94 L of N_2_; 4.86 L of N_2_ is retained in the coal body. It can be seen that N_2_ breaks through at approximately 0.5 displaced volumes.

Sweep efficiency and displaced volume are defined as follows:

(2) $$\begin{array}{c} \text{sweep}\;\text{efficiency}\;\left(\%\right)=\frac{\text{volume}\;\text{of}\;\text{injected}\;\text{displacing}\;\text{gas}}{\text{volume}\;\text{of}\;\text{C}{\text{H}}_{4}\;\text{initially}\;\text{in}\;\text{place}},\\ \text{displaced}\;\text{volume}=\frac{\text{volume}\;\text{of}\;\text{injected}\;\text{displacing}\;\text{gas}}{\text{volume}\;\text{of}\;\text{C}{\text{H}}_{4}\;\text{initially}\;\text{in}\;\text{place}}. \end{array}$$

**Figure 6:**
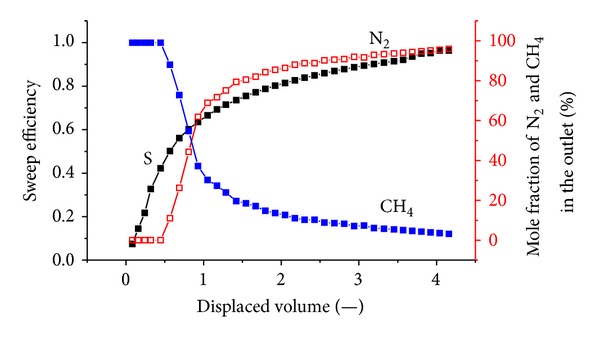
The sweep efficiency and concentrations of produced gas against displaced volume at 1.8 MPa.

Figure 6 shows the sweep efficiency and concentrations of produced gas against displaced volume at 1.8 MPa. The total volume of injected N_2_ is 37.21 L; the outlet volume of the exhaust gas is 41.1 L, including 9.24 L of CH_4_ and 31.86 L of N_2_; and 5.35 L of N_2_ is retained in the coal body, which indirectly indicates that N_2_ is more volatile and less strongly adsorbing than methane. N_2_ breaks through at approximately 0.36 displaced volumes.

**Figure 7:**
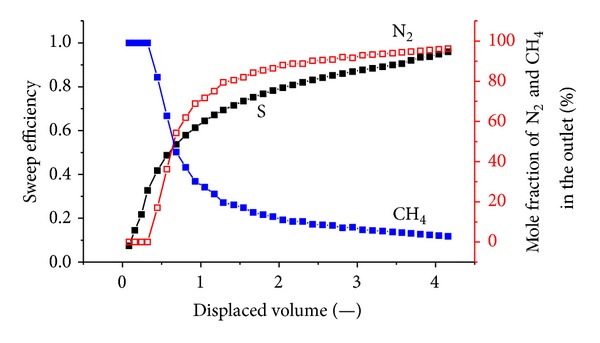
The sweep efficiency and concentrations of produced gas against displaced volume at 2.2 MPa.

Figure 7 shows the sweep efficiency and concentrations of produced gas against displaced volume at 2.2 MPa. The total volume of injected N_2_ is 36.9 L; the outlet of the exhaust gas is 40.18 L, including 9.42 L of CH_4_ and 30.76 L of N_2_; and 6.14 L of N_2_ is retained in the coal body.

#### 3.2.2. CO_2_-ECBM Experiments under Different Crustal Stresses

**Figure 8:**
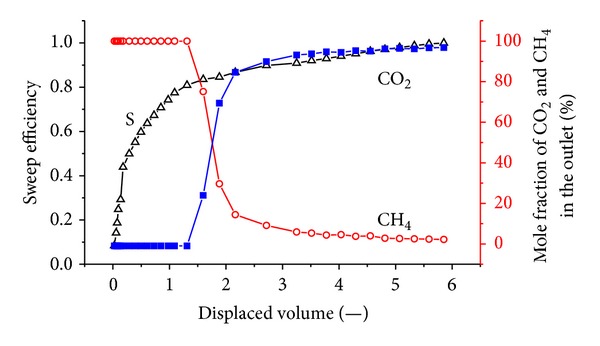
The sweep efficiency and concentrations of produced gas against displaced volume with a vertical stress of 14 MPa.

Figure 8 shows the sweep efficiency and concentrations of produced gas against displaced volume with a crustal stress of 14 MPa. The injection pressure is 1.5 MPa.

It can be seen that CO_2_ breaks through at approximately 1.4 displaced volumes. The total volume of injected CO_2_ is 43.91 L; the outlet volume of the exhaust gas is 31.89 L, including 7.5 L of CH_4_ and 24.38 L of CO_2_; and there is 19.53 L of CO_2_ retained in the coal body. The gross ratio of the CO_2_/CH_4_ displacement was approximately 2.6. At this time, the percentage of CH_4_ at the output is almost zero, indicating that the CH_4_ gas in the coal is completely displaced.

**Figure 9:**
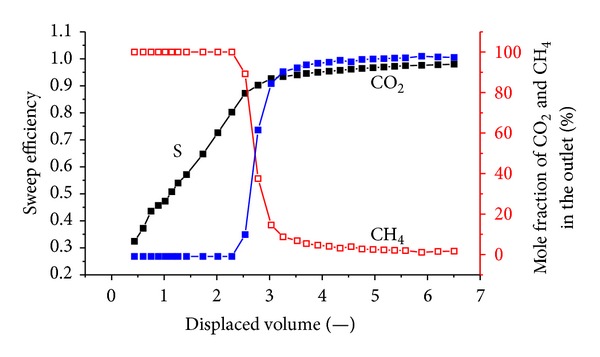
The sweep efficiency and concentrations of produced gas against displaced volume at 1.5 MPa.

Figure 9 shows the sweep efficiency and concentrations of produced gas against the displaced volume with a crustal stress of 19 MPa. The injection pressure is 1.5 MPa. The total volume of injected CO_2_ is 44.5 L; the outlet volume of the exhaust gas is 20.91 L, including 8.8 L of CH_4_ and 12.11 L of CO_2_; and there is 32.39 L of CO_2_ retained in the coal body. The gross ratio of the CO_2_/CH_4_ displacement was approximately 3.69.

#### 3.2.3. CO_2_-ECBM Experiments under Various Injection Pressures

**Figure 10:**
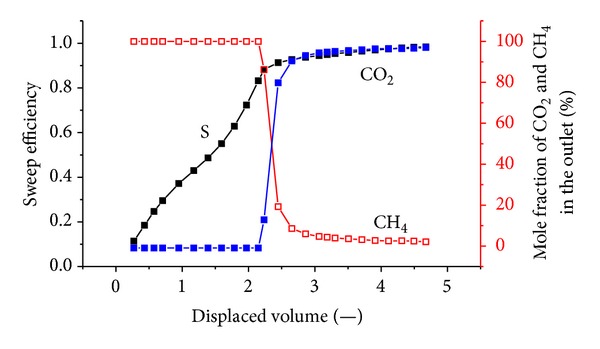
The sweep efficiency and concentrations of produced gas against displaced volume at 1.8 MPa.

**Figure 11:**
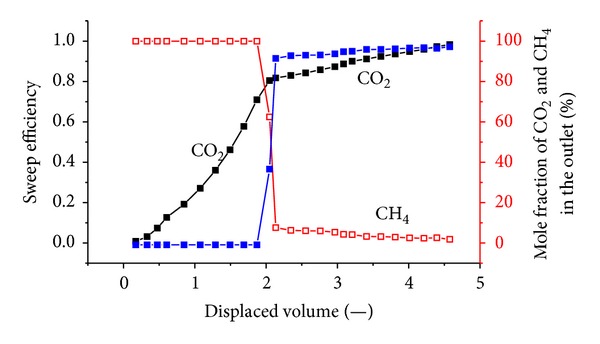
The sweep efficiency and concentrations of produced gas against displaced volume at 2.2 MPa.

In Tests 4, 5, and 6 (Table 2), CO_2_-ECBM experiments were carried out. The injection pressure is 1.5 MPa, 1.8 MPa, and 2.2 MPa, respectively, and the crustal stress is 19 MPa. The results are shown in Figures 9, 10, and 11.

Figure 10 shows the sweep efficiency and concentrations of produced gas against displaced volume at 1.8 MPa. Prior to CO_2_ injection, 9.8 L of CH_4_ was injected to achieve equilibrium. The total volume of injected CO_2_ is 44.53 L; the outlet volume of the exhaust gas is 27.73 L, including 9.52 L of CH_4_ and 18.21 L of CO_2_; and there is 26.32 L of CO_2_ retained in the coal body. CO_2_ breaks through at approximately 2.2 displaced volumes. The gross ratio of the CO_2_/CH_4_ displacement was approximately 26.32/9.52 = 2.76.

Figure 11 shows the sweep efficiency and concentrations of produced gas against displaced volume at 2.2 MPa. The total volume of injected CO_2_ is 44.72 L; the volume of the exhaust gas is 30.24 L, including 9.73 L of CH_4_ and 20.51 L of CO_2_; and there is 24.21 L of CO_2_ retained in the coal body. CO_2_ breaks through at approximately 1.9 displaced volumes. The gross ratio of the CO_2_/CH_4_ displacement was approximately 24.21/9.73 = 2.4.

## 4. Discussion

### 4.1. Influence of Gas Species on Displacement Efficiency

The results show that CO_2_ breaks through at approximately 2 displaced volumes. According to mass conservation, for flow in a porous medium, the injection gas should appear at the tube outlet after one pore volume is injected. In this case, as CO_2_ is adsorbed on the coal, the volume of CO_2_ in the free space is reduced and more than one displaced volume is required to see the breakthrough. It can also be seen that CO_2_ breaks through as a sharp front. This is due to the presence of a shock between the injection and the initial tie line.

N_2_ is more volatile and less strongly adsorbing than methane, so it travels quickly through the system, causing methane to desorb earlier than when CO_2_ is injected. More molecules of methane are desorbed for every molecule of N_2_ that is adsorbed. Therefore, volume is added to the flowing gas phase, thereby increasing the flow velocity. The N_2_ front is highly dispersed compared to the CO_2_ front in Figures 5, 6, and 7. For example, when the N_2_ concentration in the output is 3%, the sweep efficiency is 46%. However, when the N_2_ concentration increases to 50%, the sweep efficiency is 72%. There is CH_4_ in the output after N_2_ breaks through for a long time.

### 4.2. Influence of Gas Injection Pressure on Displacement Efficiency

**Figure 12:**
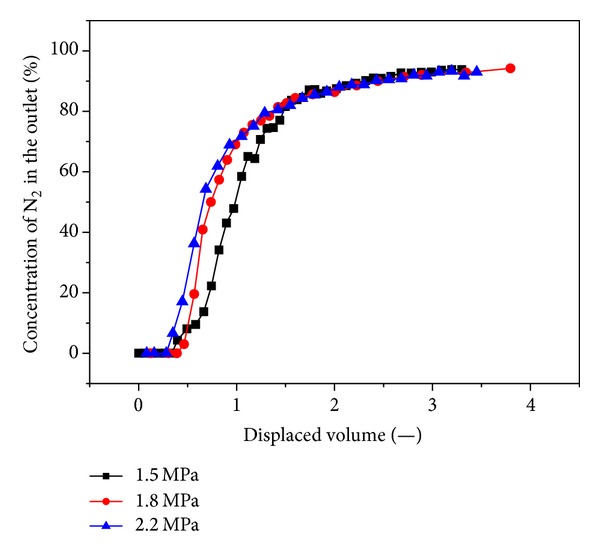
Influence on the N_2_ concentration by gas injection pressure.

N_2_ is more volatile and less strongly adsorbing than methane, so it travels quickly through the system. With a higher injection pressure, the discharge of the methane volume gradually increases, and the N_2_ content in the coal briquette also increases. This occurs because the higher injection pressure further reduces the partial pressure of CH_4_ in the coal briquette so more CH_4_ is desorbed. We found that the breakthrough time of N_2_ decreases gradually with increasing injection pressure, as shown in Figure 12.

**Figure 13:**
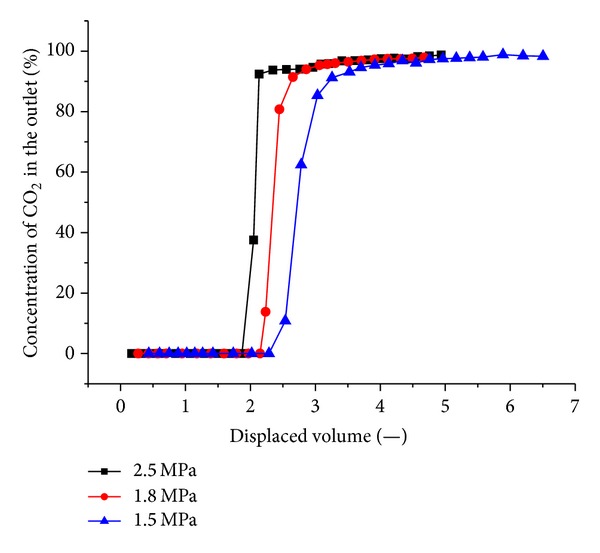
Influence on the output CO_2_ concentration by different gas injection pressures.

CO_2_ is more adsorbing and less volatile than CH_4_. When CO_2_ is injected, it is preferentially adsorbed by coal in comparison to methane. The CH_4_ is displaced. With the higher injection pressure, the adsorption of CO_2_ onto the coal increases, and more CH_4_ is desorbed.  Figure 13 shows the CO_2_ concentration profile versus the injected volume at the different injection pressures. The breakthrough time of CO_2_ was similar in all cases; however, after breakthrough, the produced CO_2_ concentration behaved somewhat differently. With high pressure, the effluent concentration increases sharply, indicating that the displacement is piston-like. When the pressure is lower, the produced CO_2_ concentration is more dispersed. Higher pressure reduces the time required for CO_2_ to displace CH_4_ from coal surfaces.

**Figure 14:**
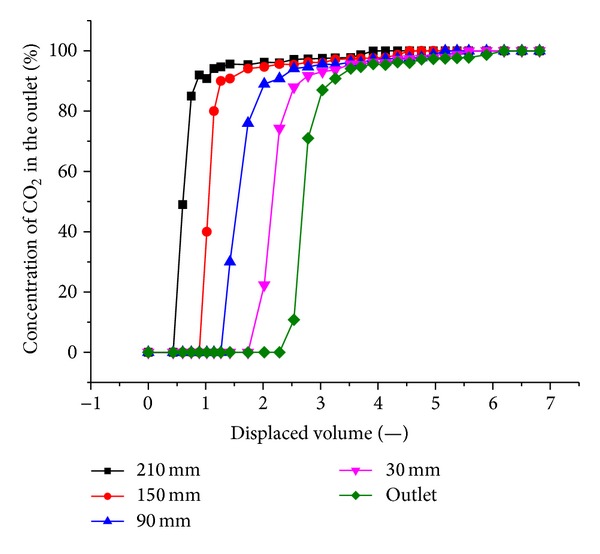
CO_2_ concentration versus time sampled from various locations along the length of the coal pack. The injection pressure of CO_2_ was maintained at 1.5 MPa.

The sampling points along the length of the coal pack allow the measurement of the composition of the free gas during the tests.  Figure 14 shows the gas composition of sampling points versus the injected volumes at 1.5 MPa injection pressure. The figure indicates that the closer the distance from the inlet, the steeper the CO_2_ concentration changes. We obtain similar curves at other injection pressures.

### 4.3. Influence of the Stress on Displacement Efficiency

When coal exhibits high permeability, the flow rate of CO_2_ in the coal is also high. The coal does not adsorb CO_2_ sufficiently, so sweeping plays an important role in the displacement process. Most of the CH_4_ is swept away by CO_2_ rather than removed primarily by replacement. Once the permeability decreases, the flow rate of CO_2_ in the coal slows. CO_2_ can then be adsorbed onto the coal, and CH_4_ is flooded out step by step under the effect of CO_2_. Therefore, the CO_2_ breakthrough time gradually slowed from 1.4 to 2.4 displaced volumes. After the CO_2_ breakthrough at the outlet, the concentration of CO_2_ soon reaches 90%.

**Figure 15:**
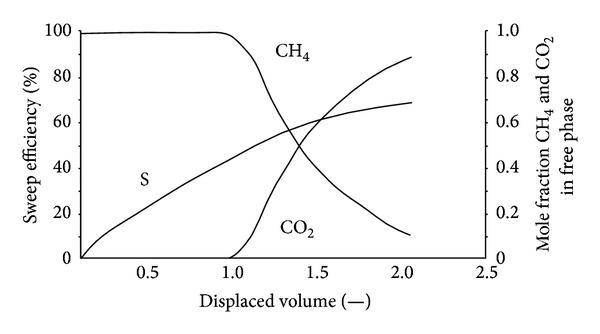
The sweep efficiency and molar concentrations of the produced gas against the displaced volume.

Mazumder et al. studied raw coal. The change in the gas concentration in the outlet is not in accordance with this experiment [15]. In this study, CO_2_ breaks through air outlet, so its concentration does not increase quickly (to more than 90%), as shown in Figure 15. This experiment uses the coal briquette made from 0.20~25 mm size of pulverized coal, so the time for diffusive exchange of gases from the particle exterior to the center of the particle is quite short. CH_4_ can quickly be desorbed from the coal matrix. In regard to intact coal, the permeability of coal is low in general. The time required for the diffusion of gas from the outside to the core is slow, leading to a slower CH_4_ desorption rate. CH_4_ can continue to spread out from a coal matrix. The results of this paper are consistent with those of Parakh [14].

### 4.4. Pore Pressure Changes Accompanying the ECBM Experiments

**Figure 16:**
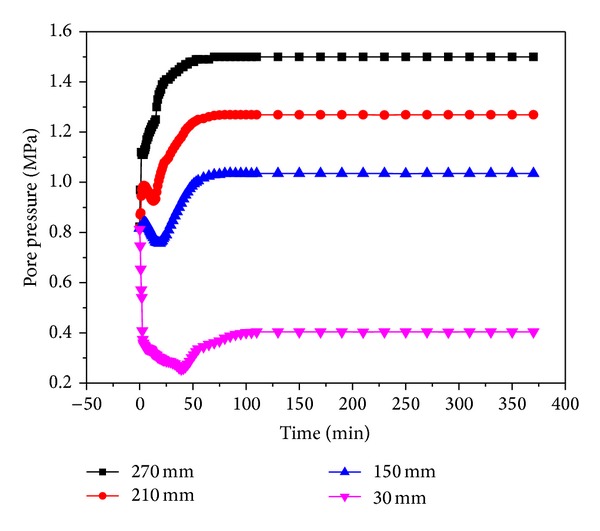
The change in pore pressure during CO_2_-ECBM at 1.5 MPa.

**Figure 17:**
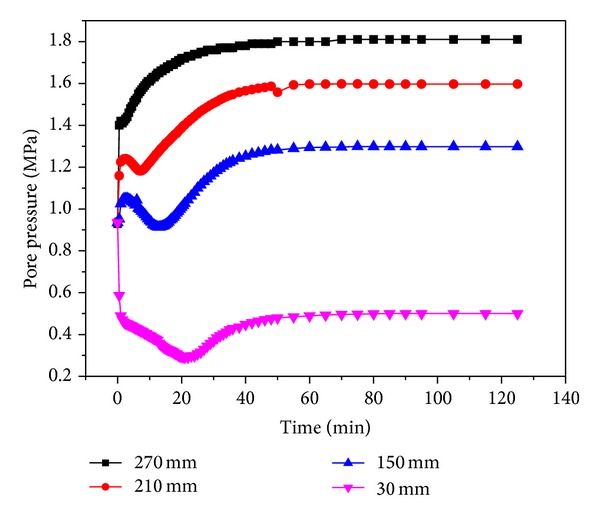
The change of pore pressure during CO_2_-ECBM at 1.8 MPa.

Figures 16 and 17 show the pore pressure changes accompanying the ECBM experiments with injection pressures of 1.5 MPa and 1.8 MPa under a crustal stress of 19 MPa. The point “30 mm” is close to the outlet, and the pressure drops the fastest here in the early stages. When CO_2_ is injected continuously, at this location the pressure is the lowest at 0.26 MPa. The pressure then increases gradually and is stable after 110 minutes. The point “270 mm” is close to the inlet and is not affected by the exhaust outlet. The pressure increases continuously to a level that is slightly lower than the injection pressure. For the other points “150 mm” and “210 mm”, the pore pressure increases over a short period of time and then reduces and increases until a stable value is finally reached. The other groups of experiments also showed a similar curve.

## 5. Main Conclusions

We have presented experimental results of two ECBM tests carried out using N_2_ and CO_2_ as injectants. The pressures at the outlet and inlet points, the gas production rate, and gas composition are reported. The main conclusions follow.

Gas injection significantly enhances CBM recovery. The experimental recoveries of the original gas are in excess of 93% for all cases. When 0.5 displaced volumes are injected, N_2_ breaks through the outlet. With increasing injection pressure, the breakthrough time shortens. N_2_ advances more rapidly and displays a more dispersed front than CO_2_, which is more adsorbing and less volatile than CH_4_. Therefore, CO_2_ breakthrough requires the injection of more than one displaced volume. At three injection pressures of 1.5 MPa, 1.8 MPa, 2.2 MPa, CO_2_ breaks through at 1.4~2.4 displaced volumes. CO_2_ moves through coal in a piston-like fashion. Once CO_2_ breaks through the outlet, the CO_2_ concentration quickly achieved high values (more than 90%). The breakthrough time of CO_2_ is reduced with increasing injection pressure.

With the increase of *in situ* stress, permeability decreases, and the seepage speed of CO_2_ slows in coal. The coal can adsorb CO_2_, which results in a slower breakthrough time. Under a ground stress of 14 MPa, CO_2_ breaks through at 1.4 displaced volumes, while at 19 MPa, the breakthrough time is approximately 2.4 displaced volumes. The ground stress significantly influences the displacement effect of gas injection.

## Figures and Tables

**Table 1:** Properties of coal sample.

Sample no.	Proximate analysis/%				Maceral/%			*R*_0_/%
	*M*_ad_	*A*_*d*_	*V*_daf_	*FC*_*d*_	Vitrinite	Inertinite	Exinite	
No. 1	1.90	7.44	27.4	67.2	60.57	38.18	0	1.09

**Table 2:** Displacement experiment conditions and the results of different pressures of gas injection.

Number	Injectant gas	Injection pressure/MPa	Displaced volume	Sweep efficiency/%
Test 1	N_2_	1.5	0.4	93.8
Test 2	N_2_	1.8	0.5	95.26
Test 3	N_2_	2.2	0.3	97.11
Test 4	CO_2_	1.5	2.4	94.44
Test 5	CO_2_	1.8	2.2	97.14
Test 6	CO_2_	2.2	1.9	98.28

**Table 3:** Displacement experiment conditions and results under different stress conditions.

Number	Stress/MPa	Injectant gas	Injection pressure/MPa	Displaced volume	Sweep efficiency/%
Test 7	14	CO_2_	1.5	1.4	97.82
Test 4	19	CO_2_	1.5	2.4	94.44
